# Quantitative Wide-Field Imaging Techniques for Fluorescence Guided Neurosurgery

**DOI:** 10.3389/fsurg.2019.00031

**Published:** 2019-06-06

**Authors:** Pablo A. Valdes, Parikshit Juvekar, Nathalie Y. R. Agar, Sylvain Gioux, Alexandra J. Golby

**Affiliations:** ^1^Department of Neurosurgery, Harvard Medical School, Brigham and Women's Hospital, Boston, MA, United States; ^2^ICube Laboratory, University of Strasbourg, Télécom Physique Strasbourg, Alsace, France

**Keywords:** fluorescence-guided surgery, quantitative fluorescence imaging, protoporphyrin IX, tissue optical properties, brain tumors

## Abstract

Fluorescence guided surgery (FGS) has fueled the development of novel technologies aimed at maximizing the utility of fluorescence imaging to help clinicians diagnose and in certain cases treat diseases across a breadth of disciplines such as dermatology, gynecology, oncology, ophthalmology, and neurosurgery. In neurosurgery, the goal of FGS technologies is to provide the neurosurgeon with additional information which can serve as a visual aid to better identify tumor tissue and associated margins. Yet, current clinical FGS technologies are qualitative in nature, limiting the ability to make accurate, reliable, and repeatable measurements. To this end, developments in fluorescence quantification are needed to overcome current limitations of FGS. Here we present an overview of the recent developments in quantitative fluorescence guidance technologies and conclude with the most recent developments aimed at wide-field quantitative fluorescence imaging approaches in neurosurgery.

## Introduction

**Figure 1:**
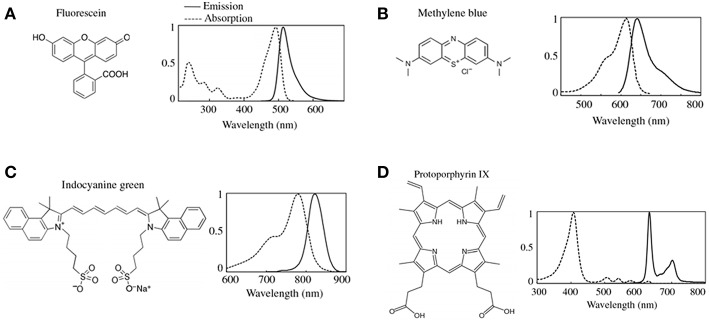
Common fluorophores in clinical use. The chemical formulas and the associated excitation and emission spectra for the most common FDA approved fluorescence dyes are shown in **(A)** fluorescein, **(B)** methylene blue, **(C)** indocyanine green (ICG), and **(D)** protoporphryin IX (PpIX). Figure adapted with permission from DSouza et al. (12).

Fluorescence guided surgery (FGS) has fueled the development of novel technologies aimed at maximizing the utility of fluorescence imaging to help clinicians diagnose and in certain cases treat diseases across a breadth of disciplines such as dermatology, gynecology, oncology, ophthalmology, and neurosurgery. In neurosurgery, FGS has been applied in a variety of diseases including high grade gliomas where the largest experience exists, but also in other brain tumors including low-grade gliomas, meningiomas, lymphomas, and metastases. In addition to the use of FGS for brain tumors, neurosurgeon have used fluorescence for vascular imaging as well (1–11). The most common fluorophores, or fluorescent biomarkers in use include 5-aminolevulinic acid (5-ALA) induced protoporphyrin IX (PpIX), fluorescein sodium, methylene blue, and indocyanine green (ICG) (Figures 1A–D). There are also a variety of novel targeted fluorescent agents being tested in clinical trials (4, 9). FGS requires the development of novel agents with the ability to map the biological properties of interest (e.g., high specificity and sensitivity for tissue) as well as accompanying intraoperative instrumentation technologies for accurate, sensitive, specific, and objective assessment of the fluorescence emitted by these biomarkers.

Exciting developments in novel technologies for FGS to treat brain tumors include new wide-field fluorescence microscopes and hand-held devices. The goal of these technologies is to provide the neurosurgeon with additional information which can serve as a visual aid to better identify tumor tissue and associated margins. Beyond implementation of 5-ALA-PpIX across multiple different pathologies (e.g., gliomas, meningiomas, metastases, CNS lymphomas, spinal tumors), quantification of fluorescence in FGS opens a new avenue of research for novel technological development. Fluorescence quantification is needed to overcome current limitations of FGS which has been qualitative in nature, limiting the ability to make accurate, reliable and repeatable measurements. These limitations, in turn, impede consensus, standardization, and adoption of FGS in the field. To this end, various technologies, both pre-clinical and clinical, have been developed which are aimed at quantification and objective means of assessment of intraoperative fluorescence. Here we present an overview of the recent developments in quantitative fluorescence guidance technologies, with a focus on 5-ALA-PpIX, and conclude with the most recent developments aimed at wide-field quantitative fluorescence imaging approaches in neurosurgery.

## Fundamental Concepts

State-of-the-art, clinically approved systems for FGS using PpIX provide surgeons with qualitative images of the “raw” fluorescence emissions as observed through the oculars of a surgical microscope modified for fluorescence imaging. During surgery, neurosurgeons can switch from conventional, white light illumination imaging mode to fluorescence light illumination mode to visualize either no visible fluorescence, or various graded, qualitative assessments of fluorescence intensities [e.g., in the case of fluorescein green-yellow (Figure 1A), or PpIX red-pink (Figure 1D)] from low to very bright fluorescence. Surgeons use these qualitative assessments of the fluorescence, herein called visible fluorescence imaging (vFI), to make clinical judgements. Neurosurgeons make qualitative assessments of the fluorescence visualized through the oculars, to ascertain the presence (or absence) of tumor (6, 7, 9, 11). PpIX emits in the 610–720 nm range when excited with 405 nm light to produce a red-pink fluorescence when visualized with state-of-the-art commercial surgical microscopes for FGS (3, 4, 11, 13) (Figure 1D). Numerous clinical studies have demonstrated a strong >90% positive predictive value of visible (e.g., bright pink fluorescence) PpIX vFI for predicting the presence tumor. As such, in areas with high or bright levels of visible fluorescence, the surgeon will make the judgement of the presence of tumor. Nevertheless, vFI assessments using state-of-the-art clinical microscopes during PpIX FGS have demonstrated a negative predictive value and sensitivity < 50% in numerous studies (4, 11). Therefore, in areas with no visible fluorescence, the surgeon will make the judgement of no tumor present. Yet, given the high false negative rate of vFI PpIX there remains a high likelihood for the presence of residual tumor.

The low negative predictive value and sensitivity of vFI with 5-ALA-PpIX noted in glioma studies sheds light on important fundamental concepts in biomedical optics and on the limits of current state-of-the-art clinical technologies (4, 14, 15). It is well-known in biomedical optics, that multiple factors come into play with respect to *in vivo* fluorescence measurements during surgery or similar applications (3, 4, 14–16). Here, we will elaborate on some of the fundamental principles to consider in the implementation of fluorescence technologies during FGS, with a focus on further needs and developments in terms of quantification, or objective measures of the fluorescence intensity. We will describe the differences between visible fluorescence imaging (vFI) and the concept of quantitative fluorescence imaging (qFI). We will make use of fundamental ideas in biomedical optics to present the key factors to consider when developing quantitative FGS technologies. After building on the fundamental biophysics of tissue fluorescence measurements, we will describe current developments and applications of qFI in neurosurgery.

### Tissue Optics

The measured fluorescence intensity, or fluorescence light that reaches the surgeon through the surgical oculars, or which reaches a detector (e.g., camera) and is displayed on a screen depends on multiple factors. These factors may be divided into instrumentation and intrinsic factors. Instrumentation factors include the specific camera properties (e.g., dark noise, pixel size, amplification, binning, etc.), light source excitation power, microscope optics (e.g., filters, mirrors, lenses), and set up (e.g., distance between excitation and tissue, distance between tissue and camera/oculars) (3, 14, 16). Here, we will not elaborate further on these components but acknowledge their significant role in our interpretation of the fluorescence and refer the reader to prior studies (12, 16–18). In the present review, we will focus on intrinsic factors impacting fluorescence, and how we can exploit understanding of these factors in developing quantitative fluorescence technologies.

#### Endogenous (Auto) Fluorescence

In the intraoperative setting, when tissues are interrogated for a fluorophore of interest (e.g., PpIX), two major intrinsic factors can impact the visualized or detected fluorescence: tissue autofluorescence (***AF***) and tissue optical properties—absorption (μ_a_) and reduced scattering (μ_s_’). Tissue ***AF*** results from endogenous fluorophores which make up cells and tissues (14). Multiple endogenous fluorophores varying by tissue composition include but are not limited to nicotinamide adenine dinucleotide (NADH), flavin adenine dinucleotide (FAD), aromatic amino acids (e.g., tryptophan), structural proteins (e.g., collagen, elastin), and degradation products (e.g., lipofuscin). These fluorophores have their excitation maxima in the range 200–400 nm and their emission maxima in the range 300–500 nm (14). As such, fluorescence imaging of tissues can have overlapping signal contributions from the fluorophore of interest (e.g., PpIX, fluorescein) and tissue ***AF***, which can lead to overestimation of fluorescence intensity from the fluorophore of interest. Thus, to properly quantify fluorescence, technologies require a means for spectral unmixing of tissue ***AF*** (and additional fluorophore contributions) from fluorescence due to the biomarker of interest such as PpIX, that make up the fluorescence measurements. For example, in the case of PpIX, photobleaching effects produce photoproducts (19–21), which can lead to inaccurate measurements of PpIX fluorescence given overlapping fluorescence emissions in the main PpIX emission peak. Other aspects to consider with fluorophores like fluorescence is fluorophore leakage from the vasculature, unlike PpIX which to the authors' knowledge, no large clinical study has noted leakage of intracellular PpIX contents as with fluorescein (4, 9, 22). Of note, current modified surgical microscopes for FGS provide surgeons with visualization of fluorescence emitted from tissues without any unmixing (e.g., subtraction) of tissue ***AF*** from the fluorescence produced by the fluorophore of interest (e.g., PpIX). These fluorescence measurements can then over or under estimate the actual fluorescence contribution from the fluorophore of interest, leading to inaccurate assessments of biomarker.

#### Tissue Optical Properties: Absorption and Scattering

**Figure 2:**
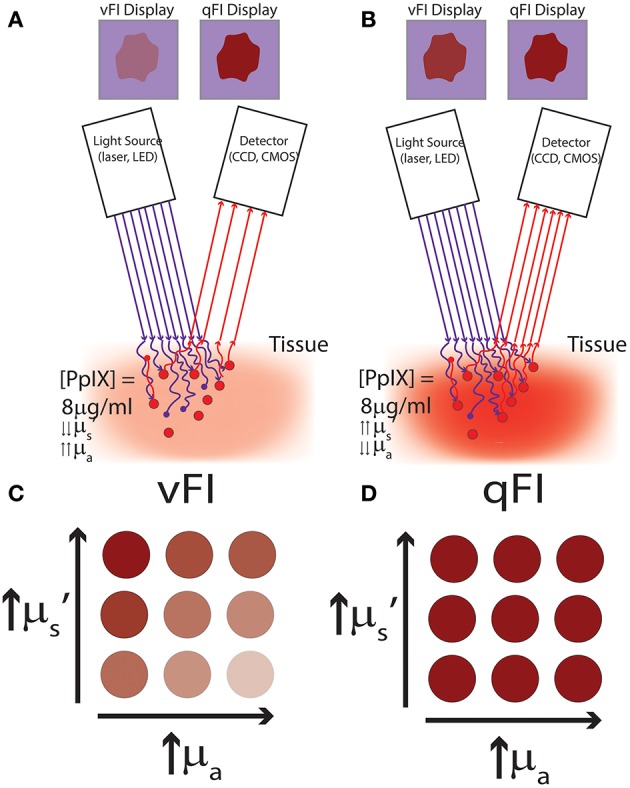
Schematics of vFI and qFI for fluorescence guided surgery. **(A,B)** show schematics of an intraoperative FGS setup, which includes a light source for excitation light (e.g., lasers, light emitting diodes), and a detector to collect the fluorescence emissions (e.g., a CCD or CMOS camera). Tissues are illuminated with 405 nm violet light to excite the fluorophore, PpIX, which emits red to far red fluorescence in the 620–710 nm range. Excitation photons reach tissue and undergo multiple interactions with tissue including absorption and scattering of photons, with only a subset of excitation photons reaching the fluorophore, PpIX. Upon excitation, PpIX emits fluorescent photons which in turn travel through tissue undergoing multiple interactions including absorption and scattering, with only a subset of these fluorescent photons exiting tissue to reach the detector. In **(A)** less excitation photons reach PpIX and less fluorescent photons exit to reach the detector in tissues with high absorption and low reduced scattering. Meanwhile, in **(B)** more excitation photons reach PpIX and more fluorescent photons exit to reach the detector in tissues with low absorption and high reduced scattering, despite both **(A,B)** containing the same concentration and distribution of PpIX fluorophore. In **(A,B)** vFI shows highly different images of the surgical field of view (upper panel labeled vFI Display) despite equal PpIX concentrations, meanwhile, qFI shows equivalent images of the surgical field of view (upper panel labeled qFI Display) by correcting for intrinsic tissue factors including tissue optical properties. Schematics of tissue simulating phantoms containing the same concentration of PpIX but with different optical properties shown in **(C,D)**, with absorption increasing from left to right and reduced scattering increasing from bottom to top. **(C)** Shows images using vFI which either over- (top left corner) or under-estimates (bottom right corner) PpIX fluorescence with different tissue optical properties, meanwhile **(D)** shows images using qFI which performs accurate estimates of PpIX concentrations by correcting for tissue optical properties.

The measured fluorescence intensity is significantly affected by tissue optical properties, which include the tissue absorption and reduced scattering at both the excitation (μ_a(x)_, μ_s_’(*m*)) and emission wavelengths (μ_a(m)_, μ_s_’(*x*)). Tissue optical properties vary with wavelength. For example, hemoglobin has an μ_a_ (absorption) peak in the ultraviolet region ~10 times greater than its absorption in the red region of the visible spectrum (units cm^−1^). Meanwhile, reduced scattering, μ_s_’, has a greater magnitude than absorption with a power law decrease as a function of wavelength. Thus, absorption and scattering will have significantly different effects on fluorescence imaging depending on the fluorophore's excitation and emission wavelengths (14, 15). Absorption and scattering in turn are determined by the biochemical composition of tissue. Absorption (μ_a_) of light in tissues is primarily determined by endogenous tissue constituents, e.g., oxy-hemoglobin and deoxy-hemoglobin, melanin, myoglobin, and water. In the brain, the most significant contributors to absorption are oxy- and deoxy-hemoglobin with their largest absorption at <600 nm (9, 14, 23, 24). As a consequence, ultraviolet to near infrared light illuminated on to tissue will travel between ~100 μm to a few millimeters before complete loss due to absorption. In the context of FGS of the brain, a practical translation of the concept of tissue absorption would entail that areas of higher vascularity will have relatively higher levels of hemoglobin compared to other areas with lower vascularity, or areas of greater hypoxia will have higher levels of deoxy-hemoglobin relative to adjacent, better perfused areas. Using optical methodologies, one can then distinguish these different areas by referring to the specific spectra (i.e., optical signatures) of oxy and deoxy-hemoglobin. With respect to fluorophores, in tissues with high absorption (Figure 2A), more excitation and emission light would be absorbed relative to tissues with low absorption (Figure 2B). Furthermore, tissue with varying scattering will further affect the amount of excitation light that reaches fluorophores for excitation, and in turn, the number of photons which exit the tissue to reach the detector. This means that tissue with the same fluorophore concentration but different absorption and scattering will encounter less excitation light (i.e., fewer photons) reaching the fluorophore to enable excitation, and thus, less emission light would exit the tissue to reach the surgeon's oculars, or detector, leading to decreased detection of fluorescence intensity (4, 9, 14, 25).

Tissue scattering results from structural changes in tissues and is significant in the so called “therapeutic window” from 600 to 1,000 nm, where tissue absorption is small compared to scattering. Tissue scattering is determined by structures such as mitochondria, collagen fiber diameters, cell size and changes in the cell environment and its structures (14). For example, areas of higher cellularity and mitochondria content (e.g., cancerous tissue) will demonstrate different scattering than normal tissue. As such, tissue scattering can be indicative of pathophysiological changes and used as a means for optical contrast between tissues. Areas with higher scattering will allow more excitation light to travel through tissues, and furthermore, more emission light to exit tissues compared to areas with lower scattering (14). Current commercial systems for FGS in neurosurgery do not measure these optical properties or account for them in their fluorescence measurements.

#### Fluorescence Transport in Tissue

Intrinsic factors include the intrinsic properties of the fluorophore of interest (e.g., PpIX) and intrinsic tissue optical properties. The interplay of these factors determines the measured fluorescence intensity from tissue, ***F*** (i.e., the “raw fluorescence” intensity/number of photons measured by a detector or seen via the surgical oculars). Fluorophores possess their own intrinsic properties irrespective of the instrumentation used which include: concentration (**C**), quantum yield (***Q***), and extinction coefficient (**μ**); such that in the absence of any tissues (or in the setting of pure diluted fluorophore) the emitted (steady state) “raw fluorescence” intensity, ***F***, is linearly proportional to the concentration of fluorophore, **C** (Equation 1) (14, 15, 19, 20).

(1) $$\begin{array}{r} F=f=vFI=C*Q*\mu \end{array}$$

In the absence of tissues (or in the setting of pure diluted fluorophores) without the wavelength-dependent varying effects of tissue optical properties (μ_a_, μ_s_’) and confounding/overlapping autofluorescence, ***AF***, from endogenous fluorophores, ***E***_***f***_, the “raw” fluorescence intensity, ***F***, is equal to the quantitative fluorescence, ***f*** (Equation 1). In this ideal state, the fluorescence intensity seen by the surgeon, [i.e., the visible fluorescence (vFI)], is linearly proportional to the concentration of fluorophore in tissue (i.e., the brighter the fluorescence the higher the concentration independent of tissue variations). Nevertheless, in the operative setting, in which the surgeon visualizes different fluorescence intensities in tissue consisting of spatially varying levels of endogenous fluorophores (and in the case of PpIX, PpIX photoproducts), ***E***_***f***_, and tissue optical properties, ***T***, the “raw” fluorescence intensity (e.g., vFI), is determined by a complex interaction of these factors (Equation 2) (14, 15, 17–21, 26–28).

(2) $$\begin{array}{r} F=f*E_{f}*T=C*Q*\mu *T \end{array}$$

During FGS of high-grade gliomas using PpIX as biomarker, the surgeon makes a qualitative assessment, i.e., vFI, of the “raw” fluorescence intensity and visually assessed what he/she sees as levels of red-pink fluorescence (no fluorescence, low fluorescence, high/bright fluorescence). The surgeon uses this information to infer the levels of tumor biomarker, i.e., PpIX, present. That is, tissues with high fluorescence implying high levels of PpIX, will be judged as containing tumor, and those without fluorescence, implying no PpIX, will likely be judged as not having tumor. Nevertheless, the “raw fluorescence” intensity, ***F***, as measured using vFI is determined by a variety of factors which include not only the PpIX concentration in tissue but also additional endogenous fluorophores, ***E***_***f***_ (e.g., NAD, FADH, PpIX photoproducts) and tissue optical properties, ***T*** [absorption (μ_a_) and scattering (μ_s_’)] that vary throughout every region of tissues in a wavelength-dependent manner (5, 8, 11, 14, 15, 17–21, 26–31). This is the critical shortcoming of FGS as currently practiced, because any assessment of the “raw fluorescence” intensity as currently practiced with vFI will be, at best, qualitative and approximate but always inaccurate regarding the true levels of fluorophore(s) present in tissue. “Raw” fluorescence intensity measurements are in reality an inaccurate estimate since it includes contributions from all these combined factors (fluorophore concentration, ***AF***, and tissue optical properties) (15). The raw fluorescence will always either over- or under-estimate the concentration of fluorescent biomarker, e.g., in glioma surgery under-estimation of fluorophore leads to the incorrect conclusion that the visualized tissue does not contain tumor biomarker, and can result in leaving significant residual tumor tissue unidentified and unresected, increasing the rate of recurrent disease and decreasing patient prognosis (7) (Figure 2C).

Inaccurate measurements of fluorescent biomarker levels in tissue has fueled the developing of tools and methods for measuring the quantitative fluorescence, ***f***, in tissues, e.g., quantitative fluorescence imaging (qFI). Quantitative fluorescence measurements are accurate and true assessments of fluorophore(s) fluorescence decoupled from the distorting effects of tissue optical properties and endogenous fluorophores contributions (15). Thus, quantitative fluorescence measurements are linearly proportional to fluorophore concentrations in tissue and Equation (3)

(3) $$\begin{array}{r} \frac{F}{E_{f^{*}}T}=f=C*Q*\mu \end{array}$$

Quantitative fluorescence measurements could provide the surgeon a means for accurate assessment of fluorescent biomarker concentrations. The use of qFI would not have gross over- or under-estimation error in fluorophores levels as seen with vFI; and measurements across surgeons and institutions would be comparable given the objective scale of concentration levels (4, 11, 13, 15, 19, 22, 29, 30, 32–38) (Figure 2D).

Here we provided an overview of fundamental concepts in understanding the role of endogenous fluorescence, tissue optical properties, and fluorescence from fluorescent biomarkers currently used for FGS. These concepts provide a framework for understanding the need for quantitative fluorescence imaging (qFI) in neurosurgery. That is, current vFI technologies provide inaccurate assessments of the tissue fluorophore levels, and technological development should be geared toward creating technologies which are quantitative in their assessments of tissue fluorescence. To accomplish the goals of qFI, technologies require a means to correct for tissue optical properties and endogenous ***AF*** in the “raw” fluorescence data. In the next section, we provide an overview of the available technologies for wide-field quantitative fluorescence imaging (qFI) for fluorescence guided neurosurgery. We elaborate on precursors to qFI such as quantitative fluorescence spectroscopy, and subsequently describe technologies and clinical implementations of qFI in neurosurgery.

## Clinical Implementation of Wide-Field Quantitative Fluorescence Guided Neurosurgery

### Quantitative Fluorescence Spectroscopy

**Figure 3:**
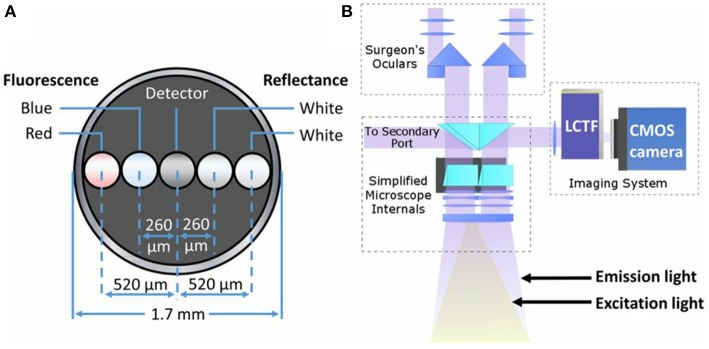
Schematics of common quantitative probe and add-on modules for quantitative fluorescence. **(A)** Schematic of the distal contact end of a quantitative fluorescence probe with a linear arrangement of fibers for white light illumination, fluorescence excitation (violet-blue channel used for surface fluorescence, red channel not used in this study), and detector fiber. **(B)** Schematic of light path set up using a commercial surgical microscope modified for fluorescence imaging with corresponding white light illumination and excitation violet-blue light. A spectrally resolved add-on system connects to a free optical port for quantitative fluorescence imaging. Figure adapted with permission from Bravo et al. (38).

To date, most clinical research implementing quantitative fluorescence assessments have used handheld spectroscopic probes (4, 9, 14, 23, 24). These probes consist of a fiber optic bundle for both light delivery and light collection with the tip of the probe held by the surgeon in contact with tissue. These probes are composed of light emitting diodes (LEDs) or laser sources to excite fluorophores and illuminate tissue, and spectrometers to collect the reflected light and/or emitted fluorescence in a wavelength-dependent manner (i.e., spectrally-resolved) (Figure 3A). A variety of spectroscopy systems have been used (4) in neurosurgery to collect fluorescence for tissue diagnostics and guidance, including probes used to collect the emitted fluorescence of endogenous (e.g., NADH, FADH), exogenously produced endogenous (e.g., PpIX), or exogenous (e.g., fluorescein) fluorophores in a wavelength dependent manner without correction for the distorting effects of tissue optical properties (5). Lin and colleagues detected *in vivo* tissue ***AF*** (i.e., without addition of exogenous fluorophores) as well as white light reflectance at 460 and 625 nm in 26 patients to distinguish normal tissue from tumor with a sensitivity and specificity of 100 and 76%, respectively. Similarly, the same group used the fluorescence peak at 500 nm as a means for identifying specific tissue pathology such as radiated tissue. In subsequent studies the authors developed discrimination algorithms with probe data to achieve sensitivities and specificities of up to 94% in distinguishing tumor from normal brain (39–44). A common theme with spectroscopy probes in neurosurgery is the use of the “raw” (i.e., arbitrary units of fluorescence) PpIX fluorescence intensity peak (5, 31, 45, 46). For example, a study by Stummer et al. (6) used a handheld spectroscopy probe to “quantify” the “raw” fluorescence at 635 nm for intraoperative diagnostic purposes. The authors used the “raw” fluorescence intensity peaks in a total of 33 patients with a receiver operating characteristic curve area under the curve of 88% using a threshold of 0.28 arbitrary units of fluorescence intensity with an associated specificity of 95% and sensitivity of 72%. This probe, like the majority in the neurosurgical literature, collect the “raw” emitted fluorescence from tissue fluorophores and the collected corresponding fluorescence spectra (i.e., wavelength dependent fluorescence light emissions). The collected “raw” fluorescence in these studies is equivalent to the “raw” fluorescence, ***F*** noted in Equation (2), which is a composite of multiple factors and thus, not truly “quantitative” measurements of the fluorescent biomarker. Nevertheless, these demonstrate the utility of improved excitation-collection geometries of contact probe-based systems. Handheld probes are in direct contact with tissue, and thus, are more efficient in both excitation of fluorophores (i.e., more light reaches the fluorophores for excitation) and collection of fluorescent emissions (i.e., more fluorescent light reaches the detector). These systems thus demonstrate the general trend of increased sensitivity compared to vFI using a modified surgical microscope (3, 14, 15, 23).

More recent developments of handheld spectroscopic probes with the aim of more accurate measurements of the true fluorophore-derived fluorescence, i.e., quantitative fluorescence, use algorithms for unmixing (e.g., subtraction) of ***AF***, multiple fluorophores, fluorescent photoproducts, or fluorophore peaks. Montcel et al. used a ratio of the fluorescent emissions collected via a handheld spectroscopic probe at 620 nm divided by the emissions at 634 nm. They corrected this ratio furthermore for tissue ***AF*** following correction of auto fluorescence as a means for more accurate spectroscopic detection (10). In similar fashion, Hosseini et al. used a spectroscopy system to “quantify” PpIX. In their work, they derived a “ratio number” for tissue diagnosis which was the ratio of the fluorescence intensity in arbitrary units at 635 nm minus the auto fluorescence at 635 nm. They then divided by the auto fluorescence at 510 nm to produce the “ratio number.” The authors reported a higher “ratio number” in tumor compared to normal brain in a limited number of patients (47). In a subsequent study, they used the ratio of the raw fluorescence intensity at 630 over 600 nm for tissue diagnosis (48). These spectroscopy studies acknowledge the importance of different fluorescent contributions to the collected, “raw” fluorescence, ***F*** (Equation 2), and developed means to correct for them in their processing of fluorescence measurements. The different algorithms account for ***AF***, ***(Ef*,** Equation 2), or for PpIX associated photoproducts in their calculations of the PpIX specific fluorescence. PpIX is known to produce distinct photoproducts and to exist in distinct photochemical states with variation in their spectra depending on factors such as pH (10, 20). This is a significant advancement in spectroscopy probes which allows spectral unmixing of major component(s) in the “raw” fluorescence, ***F***, to arrive at a more quantitative, and accurate measurement of the “quantitative fluorescence,” ***f***, and fluorescent biomarker concentrations. Fluorophores exhibit different effects to continuous light excitation, which can lead to irreversible photodamage, or fluorescence quenching, and creation of photoproducts. PpIX is more prone to photobleaching effects than some modern fluorophores used in the basic sciences such as Alexa Fluor agents or quantum dots. In the case of PpIX, multiple photoproducts have been identified with fluorescence emissions which overlap with the main PpIX peak. As such, spectral unmixing is important in accounting for not just tissue autofluorescence, but also for these confounding photoproducts which may lead to inaccurate estimates of PpIX concentrations (4, 19–21). However, these systems do not correct for the non-linear, spatially dependent, and highly variable differences in tissue absorption and scattering, ***T*** (Equation 2), and thus cannot be accurately called “quantitative fluorescence.”

Subsequent studies have used developing concepts in biomedical optics to apply correction techniques for the distorting effects of tissue optical properties with handheld spectroscopic tools (15, 26, 49–51). Correction techniques can be broadly categorized as model-based or empirical. The model-based approaches use a model of light transport (e.g., diffuse theory) to correct for tissue optical properties, usually requiring explicit measurement, and calculation of these properties prior to correcting the “raw” fluorescence spectra. Empirical models, rather than explicitly calculating tissue optical properties, will measure or calculate surrogates of these, for example, the use of reflectance measurements at distinct wavelengths. Ratiometric approaches, a common form of the latter, use ratios of the fluorescence emissions over the measured reflectance at specified wavelengths (15). Valdes et al. (33) developed a ratiometric correction approach applied to probe spectroscopy data. The authors collect the spectrally-resolved fluorescence from tissue. They then measure the reflectance near the excitation and main emission peaks; and use the calibrated reflectance correction factor to divide the “raw” fluorescence spectra to derive a “quantitative fluorescence” spectrum and quantitative PpIX concentrations (Equation 4). The “quantitative fluorescence” in this study performs well in phantoms and *in vivo* when correcting for absorption and scattering, by using the white light reflectance ratio as a surrogate for tissue optical properties effects (33) (Equation 4).

(4) $$\begin{array}{r} \frac{F}{R}=f, \end{array}$$

Valdes et al. used the same quantitative probe with a model-based correction approach to calculate the quantitative fluorescence in tissues and thus, PpIX concentrations during brain tumor resections (19, 29, 35, 36). This approach used the white light reflectance to measure the reflected white light; and using a spatially resolved model of the diffuse reflectance explicitly calculate the tissue absorption and scattering. The tissue optical properties are used in a light transport model of the fluorescence to correct the “raw” fluorescence for the distorting effects of tissue optical properties and calculate the corrected, or quantitative fluorescence (and PpIX concentrations) at each interrogated site. This group used the probe on a variety of tumor pathologies including low- and high-grade gliomas, meningiomas, and metastases demonstrating improved detection of tumor compared to vFI using commercial systems. For example, the quantitative probe was able to detect significant amounts of PpIX in low grade gliomas which were not identified using vFI, and detection accuracies in low grade gliomas were similar to those using state-of-the-art vFI for high grade gliomas. Similar to the previous approaches which accounted for endogenous or other fluorophore contributions, the authors used a spectral unmixing algorithm to account for autofluorescence, PpIX photoproducts, and PpIX (4, 19–21). These latter two approaches describe the use of a spectroscopic handheld probe similar to the previously mentioned studies. The authors collected both spectrally resolved fluorescence and diffuse white light reflectance. Similar to previous studies, they accounted for additional fluorophore contributions other than PpIX such as the endogenous autofluorescence and PpIX photoproducts. A lesson learned from these later approaches is how they correct for tissue optical properties, either by a ratiometric, and empirical approach, or by means of a model-based, light transport approach. Correction for tissue optical properties in addition to additional fluorescence contributions, enabled the authors to explicitly calculate the quantitative fluorescence and as such, PpIX biomarker concentrations for tumor tissue identification.

Hand-held spectroscopy probes informed the community regarding important factors to consider when developing quantitative technologies and more importantly, the role these measurements might play in helping improve FGS with more accurate (and at times sensitive) measurements. The different probe implementations to date used methodologies to correct for endogenous ***AF***, for fluorophore photoproducts or distinct fluorescence states, the fluorophore of interest (e.g., PpIX), and for tissue optical properties. To accomplish these tasks, spectroscopy probes acquire spectrally-resolved data to analyze the fluorescence spectra in a wavelength dependent manner, and as such, enable such analyses of spectral-fitting. Furthermore, these studies used either surrogates of tissue optical properties in the case of ratiometric approaches, or explicitly measured them using models of light transport. These probes demonstrated improved accuracies for tumor detection across a broad range of pathologies, supporting the need for technologies that are not just more sensitive, but also which perform quantitative fluorescence measurements (4, 9, 14, 23).

### Wide-Field Quantitative Fluorescence

Implementation of quantitative fluorescence systems for neurosurgical guidance is limited but included pre-clinical studies in phantoms and animals as well as clinical implementations of these novel imaging systems (3, 4, 15, 52). Yang et al. developed a multispectral fluorescence imaging system that measures fluorescence at multiple wavelengths. They tested this system during brain tumors surgeries using the agent Photofrin. This study used multi-wavelength excitation and emission light in a wide-field FGS imaging setup (53), unlike commercially available surgical microscopes which are single wavelength systems (e.g., a single bandpass for excitation and long pass filters), which limits their utility to one fluorophore at a time (3, 13). Further, as noted with the spectroscopy studies, this system is limited in its ability to correct and account for ***AF*** or additional fluorophore contributions as well as tissue optical properties on the collected fluorescence emissions. In 2011, Saager et al. (54) reported the development of a system capable of dual spatial frequency domain imaging (SFDI) and fluorescence imaging. This system performs patterned illumination at varying spatial frequencies and phases of the field of view (e.g., phantoms, animal brain) to recover the reflected light in a spectrally-resolved manner and calculate the tissue absorption and scattering at every pixel in the entire field of view (SFDI) (55). The authors were able to measure tissue optical properties to calculate a “correction map” and apply this for fluorescence correction and ultimately, quantification of PpIX concentrations. The authors validated their system in phantoms and ultimately applied to optical measurements of skin. Of note, although this study was not applied in neurosurgery or neurosurgical models, it is important because it lays the groundwork for future studies using explicit measurements of tissue optical properties for PpIX quantification. A more recent system (22) collects spectrally resolved white light reflectance and fluorescence emissions using an add-on module that adapts to a commercial surgical microscope (Figure 3B). This system collects multiple images at user-specified wavelengths in the visible range of the spectrum (e.g., 400–720 nm) enabling reconstruction of a full reflectance and fluorescence spectrum at each pixel in the image. The authors used an empiric ratiometric approach to correct for tissue optical properties by using a ratio of the “raw” fluorescence, ***F***, to the reflected white light, ***R***, to calculate the quantitative fluorescence in tissues and subsequently, using a calibration factor, calculated the true PpIX in tissues. This approach introduces important concepts in FGS. First, the authors developed a system for spectrally resolved detection of both the white light reflectance and emitted fluorescence, similar to the approach used in spectroscopy systems. Second, the authors use a fluorescence correction technique to derive the “quantitative fluorescence” in tissues. In this work, they used the reflected fluorescence as a surrogate for tissue optical properties. In the first iteration of their system, the authors used a low sensitivity, CCD camera that enabled detection levels down to ~100 ng/ml in tissue. Previous work from spectroscopic studies, noted that levels of PpIX in tumor tissues can be as low as 10 ng/ml. A subsequent system update by this team used a more sensitive scientific CMOS camera to improve both the lower threshold of detection and acquisition times by approximately one order of magnitude (19, 22). Jermyn et al developed a similar system for neurosurgical guidance using a more sensitive EMCCD camera (56). The authors noted detection levels down to 10 ng/ml of PpIX, significantly increased speeds for data collection, and improvement in overall system performance and quantification metrics compared to a CMOS based system (34). These latter studies used more sensitive, higher quality cameras, equivalent to those used for benchtop fluorescence microscopy studies, to implement into clinically compatible systems.

Xie et al. (1) developed a pre-clinical system that acquires spectrally resolved white light and fluorescence emissions, similar to Valdes et al. and Jermyn et al., and coupled it to a new algorithm for fluorescence correction. The authors developed an empirical algorithm for fluorescence correction. They correct the “raw” fluorescence by using a calibrated tissue reflectance. They calibrate their system to a known reflectance standard; and use a product of the reflectance at the excitation and emission wavelengths to subsequently correct the fluorescence spectrum. The authors tested the system in phantoms of varying absorption, scattering and PpIX concentrations to demonstrate reliable quantification <100 ng/ml with short acquisition times similar to those reported by prior studies. They noted excellent quantification (*r*^2^ = 0.94) down to <100 ng/ml in phantoms, and furthermore, provided pilot data testing of this system in *ex vivo* glioblastoma samples, demonstrating the capabilities to detect PpIX concentrations of tumor infiltrated tissues in wide-field mode. This work presents an important advancement in qFI, by developing a novel algorithm to correct for the non-linear, and confounding effects of tissue optical properties on the measured “raw” fluorescence, further demonstrating the need for techniques to measure the quantitative fluorescence, and providing a detailed account and principles for system calibration that inform the community when developing such quantitative fluorescence systems.

Sibai et al. (57) developed a bench top pre-clinical system capable of dual spatial frequency domain imaging (SFDI) and fluorescence imaging. This system performs patterned illumination at varying spatial frequencies and phases of the field of view (e.g., phantoms, animal brain) to recover the reflected light in a spectrally-resolved manner and calculate the tissue absorption and scattering at every pixel in the entire field of view (SFDI). In this study, each acquisition takes ~12 s followed by data processing. The optical properties are used to correct the collected fluorescence using a light transport model of the fluorescence; and reconstruct a qFI image of the full field of view. In this work, the authors validated their system in phantoms with varying optical properties and PpIX concentrations, and subsequently applied their system to a rodent model of glioma. They further validated their imaging results by comparison with the gold standard spectroscopy probe and *ex vivo*, tissue extraction homogenization technique demonstrating differences of μ_a_ and μ_s_’ of 14 and 19.4%, respectively and for PpIX of 10.5%. This work makes a significant advancement in quantitative fluorescence techniques in neurosurgery, by using a model-based approach for wide-field quantitative fluorescence imaging. The authors used a rigorous model-based approach for accurate estimates of tissue optical properties using a well-known SFDI technique (28). This is in contrast to the prior studies which use various calibration and empiric correction factors such as the raw reflectance as a surrogate for the tissue optical properties. After explicitly calculating the tissue optical properties across the full surgical field of view, these are integrated into a light transport model of the fluorescence to extract the quantitative fluorescence in tissue and thus, the quantitative PpIX concentrations.

**Figure 4:**
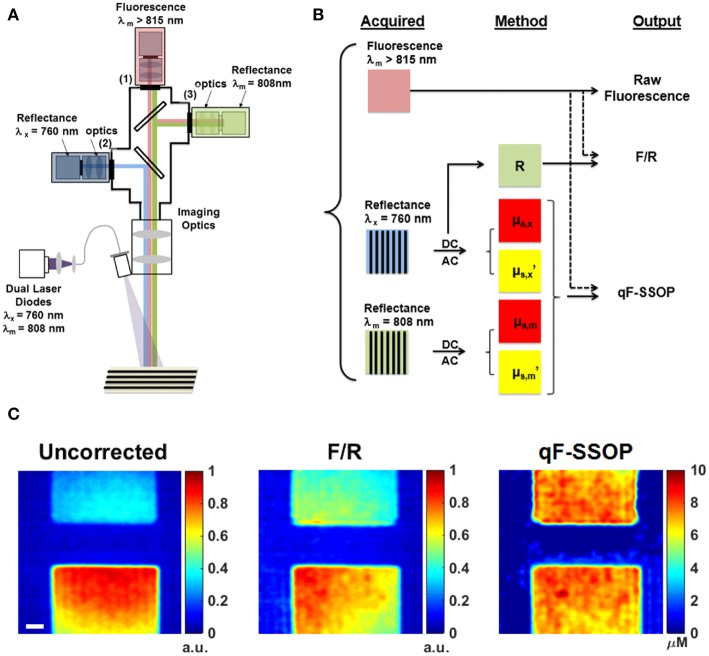
Real time, quantitative fluorescence imaging system coupled with single snapshot optical properties imaging. **(A)** Schematics of a qFI system that performs simultaneous SSOP imaging and fluorescence detection by acquiring simultaneous reflectance and fluorescence imaging under SSOP mode. **(B)** Data processing scheme in which reflectance images are processed under SSOP conditions to estimate the absorption μ_a_ and reduced scattering μ_s_’. **(C)** A light transport model is used to correct the raw fluorescence for the effects of tissue optical properties to calculate the quantitative fluorescence. Bottom panel shows one frame of a real-time dynamic video of tissue simulating phantoms displaying the raw fluorescence (uncorrected), the empirically corrected fluorescence (F/R), and the quantitative fluorescence using SSOP (qF-SSOP). Figure adapted with permission from Valdes et al. (37).

Valdes et al. (37) developed a benchtop pre-clinical system to perform simultaneous single snapshot optical properties (SSOP) imaging and fluorescence imaging in wide field mode (Figure 4A). SSOP uses only one single frequency without the need for multiple phases during patterned illumination of tissue, unlike SFDI which uses multiple frequencies and phases to extract the tissue optical properties (28, 58, 59). SSOP enables fast, real time acquisition (milliseconds per acquisition) since it requires only one image to extract optical properties compared to at least 6 required for SFDI. The authors use SSOP imaging to estimate the tissue optical properties in phantoms of varying absorption, scattering and fluorophore concentrations to correct the “raw” fluorescence using a light transport model of the quantitative fluorescence (Figure 4B). They further demonstrate the ability to perform quantitative fluorescence imaging in video rate mode as a result of the high speed offered with SSOP imaging across the full field of view in a pixel-wide manner (Figure 4C). This work provides a further development in qFI methods by using a model-based approach to estimate tissue optical properties and correct the fluorescence for these effects. Furthermore, they implement this in a wide field of mode at video rate speeds. The ability to perform video rate imaging would enable the surgeon to have immediate feedback regarding tissue in the field of view for real-time qFI.

**Figure 5:**
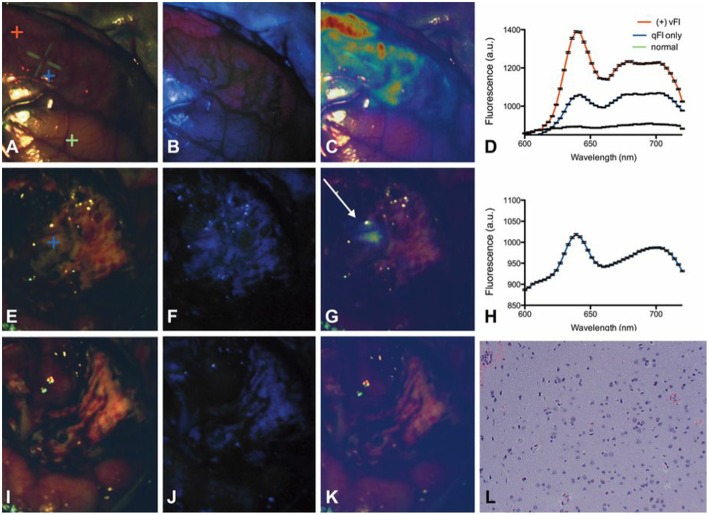
Spectrally-resolved quantitative fluorescence imaging using a ratiometric approach during *in vivo* glioblastoma surgery. Intraoperative images under **(A,E,I)** conventional white light illumination, **(B,F,J)** blue light illumination for vFI using a commercial system, and **(C,G,K)** quantitative fluorescence images using a ratiometric approach at the beginning (top row), near the end (middle row), and at the end (bottom row) of surgery. Near the end of surgery, high levels of PpIX were found using qFI **(G)** but not using vFI **(C)** with histological corroboration of tumor **(L)**. Spectra at the beginning of surgery **(D)** show expected PpIX peaks and near the end of surgery **(H)** in the area of residual tumor **(G)**. Figure adapted with permission from Valdés et al. (22).

**Figure 6:**
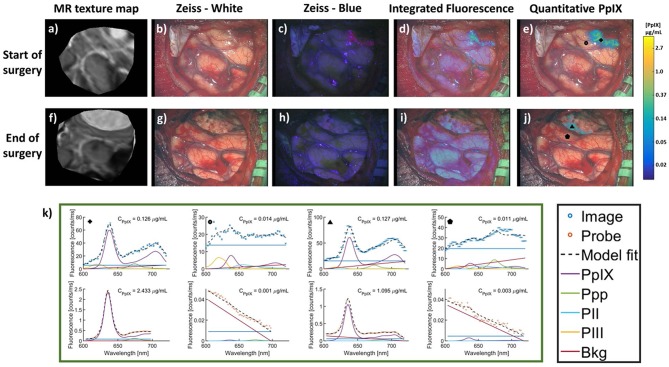
MR images and intraoperative fluorescence using a filter corrected approach. Images were acquired at the start (top row) and end (bottom row) of surgery. **(a,f)**, MR images corresponding to the intraoperative **(b,g)** conventional white light images, **(c,h)** blue light vFI images using a commercial microscope system, **(d,i)** “raw” fluorescence intensity, or integrated fluorescence images, and **(e,j)** quantitative fluorescence images using a ratiometric and filter correction approach. **(k)** PpIX spectra using the qFI system (top row) and spectroscopic quantitative probe (bottom row) at multiple locations and timepoints during surgery [marked by corresponding symbols in images **(e,j)**]. PpIX concentration observed in the C_PpIX_ overlays. Ppp, Photoproduct I; PII, Photoproduct II; PIII, Photoproduct III; Bkg, Background; Offset, linear offset for background signal. Figure adapted with permission from Bravo et al. (38).

The work above uses qFI in neurosurgical guidance in the pre-clinical setting, or on *ex vivo* human tissues. A few studies have used the above concepts and technologies and applied these in the intraoperative, clinical scenario. Valdes et al. subsequently used their spectrally-resolved, microscope add on module system in human glioblastoma surgery (22). The authors demonstrate the utility of their qFI system by showing a comparison of the images obtained using a commercially available state-of-the-art surgical microscope enabled to perform vFI and co-registered qFI images at various times points during surgery (Figures 5A–D, at the beginning of surgery; Figures 5E–G, near the end of surgery; Figures 5I–K, at the end of surgery). They demonstrate that the qFI system was able to detect tumor near the end of surgery in areas were vFI left residual tumor unidentified (i.e., vFI showed no visible fluorescence) (Figure 5F), but their qFI system was able to detect significant residual tumor (Figures 5G,H) which correlated with histopathology (Figure 5L). More recently, Bravo et al. (38) used this system *in vivo* to demonstrate the importance of spectral filtering of the fluorescence signals for more accurate PpIX quantification maps. The authors developed a metric called a “confidence ratio,” which functions as a filter to remove regions of uncertainty from quantitative PpIX images; by removing those estimates that approach the detection limits of PpIX, the authors show their approach can decrease the rates of false positives (Figure 6). This work highlights the value of performing quantitative estimates of the fluorescence to significantly improve our detection of tumor tissue across tumor pathologies compared to the standard of care using commercially available vFI technologies, and the need for complex data processing to maximize qFI detection. Furthermore, this work highlights the importance of performing wide field quantitative detection compared to single small area detection provided by handheld probes.

To date, technologies for fluorescence quantification have used either handheld, contact probes, or wide field, non-contact imaging systems. Each system boasts of their own advantages and disadvantages. Handheld probes are in direct contact with tissue, which provides a geometry for more efficient (i.e., less loss of) light excitation and collection of reflectance and fluorescence emissions from tissue. Furthermore, these systems do not have to account for different distances as they have one distance between excitation and emission sources (since they are in contact with tissue), which simplifies models for fluorescence quantification. As such, these probes have a history of algorithms developed for rigorous model-based quantification, and in more recent developments, are able to explicitly measure tissue optical properties and derive intrinsic biomarkers. These, in turn, are used in model-based approaches to correct the fluorescence emissions and quantify fluorophores. Despite the aforementioned advantages, handheld probes face a major disadvantage when it comes to wide-spread surgical implementation and intraoperative diagnostics. They require the surgeon to disrupt the surgical workflow to place the probe in direct contact with tissue, to then interrogate a small field of view (as small as 1 mm in diameter) for each acquisition (10, 14, 15, 19, 23, 31, 39, 43, 44, 47).

Wide-field, quantitative imaging systems allow the surgeons to view a larger area of interrogation up to multiple centimeters in diameter, including the full surgical field of view. This provides a more immediate, intuitive, and less disruptive view of tissue for intraoperative diagnostics. Nevertheless, current systems are limited in their ability to provide instantaneous, real-time quantification of this field of view. Another disadvantage involves a less efficient geometry for light excitation and emission given the ever-changing distances between light sources and the detector resulting from movement of the microscope and the imaging system. This, in turn, presents a challenge to accurate quantification, requiring more complex algorithms to account for these varying distances. Although, various pre-clinical systems (Table 1) have been developed that take advantage of model-based approaches for quantification (unlike systems using empiric algorithms), these have not been implemented in a seamless manner for immediate intraoperative surgical feedback. An important consideration with quantitative fluorescence is the need for calibration. Finally, spectroscopy and imaging systems require calibration against known standards of tissue optical properties and fluorophores to ensure accurate estimation of quantitative fluorescence intraoperatively (1, 4, 11, 15, 17, 22, 28, 34, 37, 38, 51, 53, 54, 56, 59). As such, reports on quantitative systems need to provide well-delineated calibration procedures to ensure translation of results between patients and institutions (18, 21, 28, 60).

**Table 1:** Comparative summary of wide-field imaging systems.

**Group**	**Fluorophore**	**Technical Features and Advantages**	**Correction Method**	**Acquisition Time**	**Processing Time**	**Cost**	**Test population**	**Limitations**
**RESEARCH SYSTEMS**								
Yang et al. (53)	Photofrin	Standalone Multispectral Fluorescence Imaging system—multiple (five band) excitation and emission wavelengths. Long working distance ~50 cm. Field of view ~ 3 cm diameter. Detector—CCD camera.	None	15 s	Real-time	~ $100,000	Phantoms. Human brain (*in vivo*.)	Inability to account and correct for ***AF, Ef***, additional fluorophore contributions and ***T***. Low concentration sensitivity (0.05 to 0.1 μg/g) and low depth sensitivity (0.5 mm.) Stand-alone system not integrated into surgical microscope. Results displayed on a personal computer.
Saager et al. (54)	5-ALA-PpIX	Standalone SFDI system—patterned illumination with varying spatial frequencies and phases of the field of view. Detector—CCD camera.	***T*** calculated as a “correction map” of the field of view using spatial frequency domain imaging	12 s	Not specified	Not specified	Phantoms. Human skin (*in vivo*).	Low concentration specificity (within 0.2 μg/ml of known concentration.) Longer acquisition times since 6 images are required to estimate ***T***. Not tested in human brains.
Valdes et al. (22)	5-ALA-PpIX Fluorescin	Integrated HIS (Hyperspectral Imaging System) for sequential spectrally resolved image acquisition in the range 400–720 nm at 10 nm intervals. Works as a portable, add-on module compatible with commercial surgical microscopes using the microscope optics and light source. Working distance ~30 cm. Field of view = 10 × 7.5 mm to 50 × 40 mm Allows reconstruction of the field of view with the full reflectance and fluorescence spectrum at each pixel as a 3D image cube. Detector—CCD camera (first iteration, concentration sensitivity ~100 ng/ml PpIX), sCMOS camera (second iteration, concentration sensitivity ~10 ng/ml PpIX.) System validated with phantoms, histopathology and comparison with commercial vFI systems.	Spectrally constrained dual-band normalization. Empiric ratiometric approach.	100 ms to 500 ms per wavelength. Up to 2–8 s per white light and fluorescence hyperspectral image capture.	Near real-time	~20,000–30,000	Phantoms. Rat brain (*in vivo*). Human brain (*in vivo*)	Penetration depth limited to a few hundred microns in depth. Quantification accuracy limited to > 40 ng/ml PpIX using the second generation sCMOS system compared to the gold standard optical probe of ~10 ng/ml PpIX. Empiric correction algorithm less accurate than model based correction approaches, unable to explicitly measure tissue optical properties and intrinsic tissue biomarkers other than PpIX
Jermyn et al. (56)	5-ALA-PpIX	Integrated HIS for sequential spectrally resolved image acquisition in the range 400–720 nm at 10 nm intervals. Works as a portable, add-on module compatible with commercial surgical microscopes using the microscope optics and light source. Working distance ~30 cm. Field of view ~ 20 cm X 20 cm. Allows reconstruction of the field of view with the full reflectance and fluorescence spectrum at each pixel as a 3D image cube. Detector—EMCCD camera (two orders of magnitude lower concentrations detected compared to CMOS, possibly comparable to the ~1 ng/ml detection limit of point probes) and sCMOS camera connected simultaneously for comparison.s	Spectrally constrained dual-band normalization. Empiric ratiometric approach.	5–100 ms. Up to 2 s per white light and fluorescence hyperspectral image capture.	Near real-time	~$50,000	Phantoms. Rat brain (*ex vivo*). Human brain (*ex vivo*)	Enhanced sensitivity of EMCCD detectors can be confounded to a greater degree by non-specific ***AF*** signals and ambient light. High cost of EMCCD detectors. Empiric correction algorithm less accurate than model based correction approaches, unable to explicitly measure tissue optical properties and intrinsic tissue biomarkers other than PpIX.
Xie et al. (1)	5-ALA-PpIX	Standalone system conceptually similar to those by Valdes et al and Jermyn et al. Detector—sCMOS camera. Concentration sensitivity ~ 10 ng/ml.	Empirical correction algorithm approach. Calibration to a known tissue reflectance standard and correction of F using a product of the R at excitation and emission wavelengths.	26 s	Not specified	Not specified	Phantoms. Human brain (*ex vivo*).	Penetration depth limited to a few hundred microns in depth. Long acquisition times due to data input/output latencies.
Sibai et al. (57)	5-ALA-PpIX	Standalone SFDI system (similar to Saager et al). Replaces previous approaches of ratiometric or semiratiometric correction in SFDI systems with a more rigorous light transport model. Allows reconstruction of the full-field of view representing qFI data post-correction. Field of view = 3 cm X 3 cm. Detector—CCD camera. System accuracy validated with spectroscopy probe (gold standard) and *ex vivo* homogenized extracted tissue. The method is also able to explicitly measure tissue optical properties and intrinsic tissue biomarkers unlike the systems by Valdes et al, Jermyn et al, and Xie et al.	Light transport model based approach using spatial frequency domain imaging.	36 s	Not specified	Not specified	Phantoms. Rat brains (post mortem in situ, *ex vivo*).	Benchtop preclinical system. Suboptimal results from *in vivo* rat brains (but these limitations may not translate to *in vivo* human experiments as the authors believe the rat glioma model to be the cause, rather than failure of the method or instrumentation). Less sensitive than spectroscopy probes by 2–4 times on account of spill-over or cross talk between image pixels and more tissue distortion and attenuation of weak fluorescence signals. Decreased concentration sensitivity and higher exposure times compared to systems with EMCCD detectors. Penetration depth limited to a few hundred microns in depth. Long acquisition times requiring 6 images to estimate T.
Valdes et al. (37)	5-ALA-PpIX	Standalone qF-SSOP system—single snapshot of optical properties. Able to perform real-time data acquisition since it requires a single image to estimate T (compared to the SFDI systems by Saager et al and Sibai et al which require multiple images).	Light transport model based approach using single snapshot of optical properties imaging	500 ms	21 ms	Not specified	Phantoms.	Benchtop preclinical system. System has not been validated against *ex vivo* or *in vivo* rodent or human brains.
Zeiss Surgical Microscope + Blue 400 module	5-ALA-PpIX	Commercial vFI system as an integrated add-on module for the Zeiss series of surgical microscopes. Real-time visualization of fluorescence through surgical oculars and projected unto a CCD camera.	No correction	Real-time	No processing	Quote requested	Humans (*in vivo*)	Requires purchase of expensive proprietary accessory. Qualitative, subjective information relayed to the surgeon without quantification. High rate of false negatives using PpIX.
**COMMERCIAL SYSTEMS**								
Leica Surgical Microscope + FL400 module	5-ALA-PpIX	Commercial vFI system as an integrated add-on module for the Leica series of surgical microscopes. Real-time visualization of fluorescence through surgical oculars and projected unto a CCD camera.	No correction	Real-time	No processing	Quote requested	Humans (*in vivo*)	Requires purchase of expensive proprietary accessory. Qualitative, subjective information relayed to the surgeon without quantification.

## Conclusion

The field of quantitative fluorescence in neurosurgery, with implementation of spectroscopic and wide-field systems is in its infancy. The majority of the literature on fluorescence imaging and its application to neurosurgical guidance in brain tumors uses vFI technologies (3). We have described limitations of vFI including subjectivity and inaccuracy of measurements, inter-observer dependence, and decreased sensitivity for residual disease. This work seeks to first introduce the reader to fundamental concepts in quantitative fluorescence, including concepts of autofluorescence, tissue optical properties and their effects on the fluorescence measurements, and fluorescence correction techniques (14, 15). Second, this study seeks to provide the reader with an overview of the major implementations of quantitative fluorescence in neurosurgery. Since the literature on quantitative fluorescence in neurosurgery is limited, we provide an overview of some of the preliminary studies seeking to arrive at quantitative assessments of the fluorescence in neurosurgery. We then highlight some lessons learned from each of these studies. Finally, we wish to inform the reader regarding the importance of quantitative fluorescence in neurosurgery both as a means for standardizing measurements across surgeons, but also, as a means for improved detection of residual disease.

The success of vFI has helped fuel technological developments including modified surgical microscopes for fluorescence imaging, exoscopes, and probe-based technologies such as spectroscopic, and confocal systems (3, 4). Furthermore, the success of vFI has subsequently led to development of quantitative fluorescence technologies discussed in this paper, as these technologies have highlighted the intrinsic limitations of vFI (subjectivity, inter-observer dependence, inaccurate measurements, decreased sensitivity for residual disease). Future developments in FGS require that these technologies provide seamless integration to the surgical workflow with fast acquisition times and ease of interpretation of the data to the surgeon. Technologies should provide means for improve visualization, calibration, and heads up display, to enable wide spread use across multiple centers. In summary, the use of qFI in neurosurgery is limited, but continued research and development will provide the neurosurgical community with more accurate technologies to ultimately improve patient outcomes.

## Author Contributions

All authors listed have made a substantial, direct and intellectual contribution to the work, and approved it for publication.

### Conflict of Interest Statement

PV reports multiple patents on optical spectroscopy and fluorescence imaging technologies. The remaining authors declare that the research was conducted in the absence of any commercial or financial relationships that could be construed as a potential conflict of interest.
